# Determinants of Physical Activity Engagement Among Male Adolescents in Riyadh, Saudi Arabia: A Comparative Study of Athletes and Non-Athletes

**DOI:** 10.3390/bs16050789

**Published:** 2026-05-15

**Authors:** Abdulrahman I. Alaqil, Fahad Bin Radhyan

**Affiliations:** 1 Department of Physical Education, College of Education, King Faisal University, Al-Ahsa 31982, Saudi Arabia; 2 Department of Physical Education, College of General Studies, King Fahd University of Petroleum and Minerals, Dhahran 31261, Saudi Arabia; fahader@kfupm.edu.sa

**Keywords:** physical activity, adolescents, motivation, barriers, obesity, Saudi Arabia, Self-Determination Theory

## Abstract

**Background**: Physical inactivity among Saudi Arabian adolescents is a critical public health concern due to its contribution to the rising prevalence of overweight, obesity, and non-communicable diseases. Despite this, the motivational profiles and perceived barriers that differentiate athletic from non-athletic adolescents remain understudied in the Saudi literature, particularly within the school Physical Education (PE) context. Grounded in Self-Determination Theory (SDT), the present study examined the factors preventing and motivating Saudi adolescents to engage in physical activity (PA) and discusses findings in terms of their implications for PE teachers and school-based intervention. **Method**: A cross-sectional study was conducted with 124 male high school students in Riyadh (mean age: 16.79 ± 0.66 years). Participants were categorized as either athletes (*n* = 70) or non-athletes (*n* = 54) based on pre-defined engagement criteria: athletes were required to report vigorous-intensity sport participation on three or more days per week for a minimum of 60 min per session. Anthropometric measurements, lifestyle behaviors (diet, screen time, sleep), motivations, and barriers were assessed using the validated Arab Teens Lifestyle Study (ATLS) questionnaire. Independent samples *t*-tests and chi-square tests were used to compare between-group differences; effect sizes are reported. **Result**: Non-athletes had a significantly higher mean BMI (29.40 ± 6.77 kg/m^2^) and waist circumference (98.65 ± 21.63 cm) compared to athletes (BMI: 22.19 ± 4.44 kg/m^2^; waist: 78.84 ± 9.51 cm; both *p* < 0.001). No significant differences were observed in screen time, sleep duration, or dietary habits. The primary motivations for PA among athletes were health benefits (27.1%), recreation (25.7%), and competition (20.0%), reflecting an autonomous motivational profile consistent with SDT. Among non-athletes, the predominant barriers were the lack of suitable facilities (25.9%) and the absence of an exercise partner (22.2%); reflecting unmet SDT needs for competence and relatedness respectively, while only 9.3% cited having a lack of time. **Conclusions**: Non-athletic participants face a significant health disadvantage characterized by higher rates of overweight and central obesity. In contrast to global trends, where academic commitments dominate barriers to PA, the principal barriers in this population are environmental and social, reflecting unmet psychological needs that PE teachers are uniquely positioned to address. Rather than focusing solely on infrastructure, PE promoters should implement need-supportive teaching practices, including competence-building tasks and cooperative peer structures, to foster the intrinsic motivational profile observed in the athletes and promote long-term PA adherence among non-athletic students, in alignment with the health objectives of Saudi Vision 2030.

## 1. Introduction

Regular physical activity (PA) is a critical determinant of adolescent health: it protects against the onset of non-communicable diseases and fosters psychological well-being and social development (Biddle et al., 2019; World Health Organization, 2020). The World Health Organization (WHO) recommends that adolescents engage in an average of 60 min of moderate-to-vigorous physical activity (MVPA) daily to achieve the above health benefits (World Health Organization, 2020). However, global adherence to these guidelines remains low, with a precipitous decline in PA levels often observed during the transition from childhood to adolescence (Al-Hazzaa, 2018; Strain et al., 2024). Within formal education systems, the Physical Education (PE) class represents one of the few structured, universal opportunities to counteract this decline, making the school environment a critical setting for PA promotion among adolescents (Cairney et al., 2019; Lounsbery & McKenzie, 2015).

In Saudi Arabia, this issue is particularly acute. A recent comprehensive scoping review highlighted that approximately 80–90% of Saudi children and adolescents fail to meet the WHO’s recommended PA levels, placing them at heightened risk of obesity and cardiovascular diseases (Evenson et al., 2023). Al-Hazzaa (2018) further emphasizes that physical inactivity is one of the primary modifiable risk factors for non-communicable diseases in Saudi Arabia, necessitating urgent public health interventions aligned with the Quality-of-Life goals of Saudi Vision 2030 (Al-Hazzaa, 2018). Critically, the school PE environment has been identified as a strategic lever for addressing this crisis, as it provides equitable access to structured PA regardless of students’ socioeconomic background or access to private sports facilities (Sallis et al., 2000).

Addressing physical inactivity in adolescents requires an understanding of specific barriers preventing engagement in PA, particularly within the school context. Such barriers tend to be multifaceted and are generally categorized into internal dimensions (e.g., lack of interest, low self-efficacy) and external dimensions (e.g., lack of facilities, safety concerns) (Gunnell et al., 2015; Musaiger et al., 2013). In the Saudi context, these barriers are further compounded by environmental and sociocultural factors (Al-Nuaim et al., 2024; Khalaf et al., 2013). Musaiger et al.’s (2013) cross-cultural study of Arab adolescents identified lack of time and a lack of facilities as the main barriers to PA participation (Musaiger et al., 2013). This concurs with the results of Alsubaie and Omer (2015), who noted that male adolescents in Riyadh frequently cite lack of time and insufficient parental support as key constraints to engaging in PA (Alsubaie & Omer, 2015). Importantly, many of these external barriers, including inadequate facilities and the absence of exercise partners, are precisely the structural conditions that school-based PE programs are uniquely positioned to address by providing supervised spaces, equipment, and structured peer interaction (Dobbins et al., 2013).

Self-Determination Theory (SDT) offers a particularly powerful framework for understanding how the PE environment can address these barriers and foster sustained PA engagement (Deci & Ryan, 2000). SDT posits that autonomous motivation, grounded in enjoyment, personal interest and internalized values, is a stronger predictor of long-term behavioral adherence than controlled motivation driven by external pressure or guilt (Dishman et al., 2015). Censoriously, SDT identified three core psychological needs whose satisfaction is essential for autonomous motivation to develop: competence (feeling capable and effective), autonomy (experiencing a sense of volition), and relatedness (feeling connected to others) (Deci & Ryan, 2000). A study by Dishman et al. (2015) confirms that autonomous motivation is a strong positive predictor of PA among middle school students, while Ullrich-French et al. (2013) demonstrated that motivation tends to shift from intrinsic to more controlled or external forms during the transition to university (Dishman et al., 2015; Ullrich-French et al., 2013). This developmental trajectory, corroborated by Sevil et al. (2018), suggests that adequately supportive environments, particularly need-supportive PE settings, inactive adolescents are at considerable risk of becoming inactive adults (Sevil et al., 2018). The PE teacher, therefore, occupies a pivotal role: by designing need-supportive learning environments, teachers can directly nurture students’ competence through skill-building, foster relatedness through cooperative activities, and support autonomy through choice and meaningful participation (Ntoumanis, 2001; Standage et al., 2005).

Unlike the Health Belief Model, which focuses primarily on perceived threat and barriers to behavior initiation, or the Social Ecological Model, which emphasizes multilevel environmental influences without directly addressing motivational processes (Campbell, 2025; Alyafei & Easton-Carr, 2026), SDT is uniquely suited to this study because it simultaneously explains both why adolescents engage in PA and what psychological conditions sustain that engagement over time. Similarly, while the Multi-Theory Model integrates multiple behavioral change constructs, SDT’s three core psychological needs, competence, autonomy, and relatedness, provide a parsimonious and empirically well-supported framework for understanding the motivational profiles and perceived barriers identified in the current study (Deci & Ryan, 2000; Ntoumanis, 2001).

Despite growing recognition of PE environment’s potential, few studies have specifically examined motivation and barriers to PA among male adolescents in Riyadh through an SDT lens. Although the prevalence of sedentary behaviors among Saudi youth is well documented (Al-Hazzaa, 2018; Begum et al., 2022; Evenson et al., 2023), there is scant comparative data on how SDT-relevant motivational profiles and perceived barriers differ between athletic and non-athletic subgroups, and fewer still have considered how the PE teacher can respond to these differences in practice. Antunes et al. (2024), which found that students involved in extracurricular sports demonstrated significantly higher achievement- and improvement-oriented motivation than those participating solely in PE classes, while Muhyi et al. (2024) reported that structural barriers such as a lack of facilities disproportionately affect adolescents not engaged in regular sports, barriers that PE teachers may be uniquely placed to mitigate within the school setting (Antunes et al., 2024; Muhyi et al., 2024).

To address this gap, the present study investigated the determinants of PA engagement among male adolescents aged 16–17 years in Riyadh, Saudi Arabia. Specifically, it sought to identify the primary motivations and barriers to regular exercise and compare anthropometric characteristics and lifestyle behaviors between athletic and non-athletic subgroups. By interpreting findings through the lens of SDT and the school PE context, it is hoped that the results will inform practical, targeted, context-sensitive strategies for PE teachers to encourage non-athletic Saudi adolescents to adopt more active, healthier lifestyles, thereby reducing the burden of preventable illness and contributing to health objectives of Saudi Vision 2030.

## 2. Materials and Methods

A cross-sectional study design was used to collect data in Riyadh, Saudi Arabia. Riyadh was selected as it is Saudi Arabia’s most populous capital, with a fast-paced urban lifestyle and greater school sports infrastructure. The sample (*n* = 124) comprised male high school students aged 16–17 years old. A priori power analysis conducted using G*Power (version 3.1.9.7) indicated that a sample of 124 participants was sufficient to detect a medium effect size (d: 0.50) with 80% power at a two-tailed significance level of α = 0.05 for independent sample *t*-test. Based on their participation in PA, the sample was divided into two groups: athletes (*n* = 70) and non-athletes (*n* = 54). Athletes were defined as students who self-reported engagement in vigorous-intensity sport or structured physical training on three or more days per week, a threshold consistent with established criteria in adolescent PA studies (Benavente-Marín et al., 2024; Sitko et al., 2025; Vella et al., 2014); participants not meeting this threshold were classified as non-athletes. Ethical approval was obtained from King Saud University Institutional Review Board (Reference: KSU-HE-22-891), and all procedures were conducted in accordance with the Declaration of Helsinki. Written informed consent was obtained from all participants following a full explanation of this study’s purpose and procedures.

### 2.1. Anthropometry

Standing height was measured to the nearest 0.1 cm using a calibrated stadiometer (Detecto Electronic, Model DHRWM, Webb City, MO, USA). Body mass was recorded to the nearest 0.1 kg using a calibrated digital scale (Seca-869, Hamburg, Germany). Body mass index (BMI) was calculated as body mass in kilograms/height in meters squared (kg/m^2^). Waist circumference (WC) was measured twice to the nearest 2 mm at the midpoint between the lower costal margin and the iliac crest using a non-stretchable measuring tape (Seca 201, Hamburg, Germany), with the mean of the two measurements used for analysis (Guilherme et al., 2015).

### 2.2. Study Questionnaire

Data on lifestyle variables were collected using the Arab Teen Lifestyle Study (ATLS) questionnaire, a validated instrument designed to assess health-related behaviors among adolescents and young adults (Al-Hazzaa & Musiager, 2011). This consists of 47 items covering domains such as physical activity patterns, sedentary behaviors, sleep duration, and dietary habits. Previous studies have confirmed the reliability and validity of the ATLS questionnaire for assessing lifestyle behaviors among those aged 14–25 years. While the ATLS was not originally designed to measure SDT constructs, the motivation and barrier items used in this study map conceptually onto SDT’s core psychological needs; the barrier ‘no suitable place’ aligns with unmet competence needs, ‘having no partner’ reflects unmet relatedness needs, and motivations such as ‘health’ and ‘recreation’ correspond to identified and intrinsic regulation in SDT’s motivational continuum. Accordingly, SDT is applied in this study as a post hoc interpretive lens rather than a prospective design framework.

#### 2.2.1. Sedentary Behavior

Sedentary behavior data were gathered using screen time as a proxy. Participants were asked to estimate their total screen time (watching television/videos or using a computer/internet, including social media). Estimates were recorded separately for school days (Sunday–Thursday) and weekend days (Friday–Saturday). These estimates were recorded separately for school days and weekend days using an 8-point ordinal scale (e.g., I do not watch TV or use a device (1) to More than 6 h (8)). For statistical analysis, these ordinal responses were converted into continuous variables representing hours per day, using the midpoint of each interval.

#### 2.2.2. Sleep Duration

Sleep pattern data were gathered by asking participants to report the average number of hours they slept per day during school days and weekend days. Responses were recorded on an 8-point scale ranging from 3 h or less to 10 h or more. Similar to screen time, these responses were converted into approximate hours for comparative analysis.

#### 2.2.3. Dietary Habits

Dietary habits data were collected using a frequency-based questionnaire. Participants were asked to report on the weekly frequency with which they consumed specific food items, with a focus on having breakfast at home and fast-food intake (e.g., burgers, shawarma). Responses were recorded on an 8-point scale ranging from ‘I never eat it’ to ‘7 times a week (daily)’. For comparison between groups, responses were coded numerically as frequencies per week (e.g., Daily = 7, Twice = 2).

#### 2.2.4. Motivations and Barriers

Data on motivating factors for PA participation and perceived barriers to PA participation were collected using specific closed-ended items from the ATLS questionnaire. Motivation was determined by using the item: If you practice physical activity regularly, what are your main reasons? Response options included health, weight loss, socializing, recreation, and competition. Barriers to PA were identified using the item: If you do NOT practice physical activity regularly, what are the reasons? Response options included lack of time, not convinced of its importance, no suitable place, health reasons, fear of criticism, and having no partner.

#### 2.2.5. Statistical Analysis

All analyses were conducted using IBM’s SPSS Statistics Package (version 29.0, IBM Corp., Armonk, NY, USA). A two-tailed significance threshold of *p* < 0.05 was applied throughout. BMI was calculated as weight (kg)/height squared (m^2^). Ordinal variables for screen time and sleep duration were recoded as continuous variables (hours per day) for parametric testing; dietary frequency items were recoded to represent the number of days per week (range: 0–7). Normality was assessed using the Shapiro–Wilk test. Descriptive statistics are presented as means ± standard deviations (SD) for continuous variables, and as frequencies (*n*) and percentages (%) for categorical variables. Between-group differences were examined using independent sample *t*-tests for normally distributed continuous variables and Pearson chi-square (χ^2^) tests for categorical variables (motivations and perceived barriers). Effect sizes were calculated using Cohen’s d for *t*-tests and Cramér’s V for chi-square analyses to assess the practical significance of findings.

## 3. Results

### Participant Characteristics

The sample (*n* = 124; mean age: 16.79 ± 0.66 years) consisted of male Saudi adolescents. Based on their self-reported PA engagement, the sample was then divided into non-athletes (*n* = 54) and athletes (*n* = 70). There was no significant difference in age between the groups (*p* = 0.753).

Independent *t*-tests revealed significant differences across all anthropometric variables except age (see Table 1 below). Athletes were significantly taller than non-athletes (*p* = 0.002). Non-athletes had significantly greater body mass (*p* < 0.001), BMI (*p* < 0.001), and waist circumference (*p* < 0.001). The non-athlete group’s mean BMI of 29.40 ± 6.77 kg/m^2^ falls within the overweight classification, whereas the athlete group’s mean BMI of 22.19 ± 4.44 kg/m^2^ falls within the normal weight range. The mean waist circumference of non-athletes (98.65 ± 21.63 cm) exceeded the clinically established threshold associated with increased cardiovascular risk, while that of athletes (78.84 ± 9.51 cm) remained within the healthy range.

No statistically significant differences were observed between groups in screen time, sleep duration, or dietary habits (all *p* > 0.05) (see Table 2 below). Although athletes reported numerically higher fast-food consumption than non-athletes, this difference did not reach statistical significance (*p* = 0.063).

Among athletes, the primary motivations for PA participation were health (27.1%), recreation (25.7%), and competition (20.0%) (see Table 3 below). Among non-athletes, the most frequently cited barriers to PA engagement were a lack of a suitable exercise facility (25.9%) and an absence of an exercise partner (22.2%), while only 9.3% cited a lack of time as a barrier (see Table 4 below).

## 4. Discussion

This study investigated the determinants (motivations and barriers) of PA engagement among athletic and non-athletic adolescents in Riyadh, Saudi Arabia, by comparing data on anthropometrics, lifestyle behaviors, and psychosocial factors between the two groups. The findings reveal significant health disparities and identify structural and social factors, rather than individual-level time constraints, as the predominant barriers to PA among non-athletic Saudi adolescents.

The most clinically significant finding was the substantial between-group difference in BMI and waist circumference. Non-athletes had a mean BMI consistent with being overweight (29.40 kg/m^2^), while athletes maintained a normal BMI (22.19 kg/m^2^), consistent with previous studies documenting the rising prevalence of overweight and obesity among Saudi adolescents attributable to sedentary lifestyles (Al-Hazzaa, 2018; Al-Hazzaa et al., 2012; Salman, 2018). Non-athletes’ mean waist circumference (98.65 cm) exceeded established thresholds associated with increased cardiometabolic risk, whereas the athlete group remained well within the healthy range (78.84 cm). Central adiposity, rather than BMI alone, is widely recognized as a stronger independent predictor of metabolic syndrome and cardiovascular risk (Alaqil et al., 2022; Guilherme et al., 2015; López-González et al., 2016; Ma et al., 2013; Yap et al., 2006), suggesting that non-athletic adolescents may face an elevated risk of developing non-communicable diseases in early adulthood. It should be noted, however, that widely used waist circumference thresholds for cardiometabolic risk (e.g., >94 cm) were derived from adult populations, and age- and sex-specific percentile-based standards should ideally be consulted when interpreting these findings in an adolescent context. These anthropometric disparities suggest the potential value of school-based interventions that embed structured, inclusive PA opportunities within the compulsory school day, where they may reach non-athletic students least likely to be physically active outside school.

Contrary to expectations, no statistically significant differences were observed between groups for screen time, sleep duration, and dietary habits. Three possible explanations may account for this. First, the near-ubiquitous availability of digital devices in Riyadh may produce uniformly high screen time rates irrespective of PA status (Alshoaibi et al., 2023; Begum et al., 2022). Second, the relatively modest sample size in the current study (N = 124) may have limited the statistical power to detect smaller between-group differences. Third, organized sports participation may occupy a discrete portion of athletes’ time without displacing screen-based sedentary behaviors during evenings and weekends. The finding that fast-food consumption was higher among athletes (*p* = 0.063) further underscores the dissociation between PA and diet in this population, highlighting the importance of integrating nutritional education into PA promotion programs rather than treating physical and dietary health as independent priorities (Sousa et al., 2024).

Among athletes, the dominant motivating factors for PA were health (27.1%), recreation (25.7%), and competition (20%). Interpreted through SDT, health and recreation represent identified and intrinsic forms of regulation, sitting toward the autonomous end of the motivational continuum, and are associated with sustained PA adherence (Deci & Ryan, 2000; Dishman et al., 2015). This suggests that athletes in this sample may have successfully internalized the personal value of PA, facilitated by environments that satisfy the psychological needs of competence, autonomy, and relatedness (Ntoumanis, 2001; Standage et al., 2005). The ranking of competition third (20%) warrants interpretive caution, as it likely reflects characteristics of those within the athlete subgroup who engage in competitive team sports rather than the group as a whole (Antunes et al., 2024). For PE teachers, this motivational profile offers a possible blueprint: prioritizing health-and enjoyment-based learning experiences over completive performance outcomes may be more likely to foster long-term PA engagement across the full student population.

The most practically significant findings related to the perceived barriers among non-athletes. Unlike Western and Latin American contexts where academic workload dominates (Allison et al., 1999; Pandolfo et al., 2016), structural and social barriers prevailed here: 25.9% cited lack of a suitable facility and 22.2% cited the absence of an exercise partner, while only 9.3% reported lack of time. Within an SDT framework, the absence of suitable exercise facilities may be associated with reduced perceived competence, the capacity to feel effective in a physical activity context, while the absence of an exercise partner reflects an unmet need for relatedness, the fundamental need to feel socially connected (Deci & Ryan, 2000; Ntoumanis, 2001; Ryan & Deci, 2017). This finding aligns with Alsubaie and Omer (2015), who highlighted the importance of environmental accessibility and peer influence in shaping PA engagement in comparable regional context (Alsubaie & Omer, 2015). From a PE perspective, the setting may be well positioned to address both simultaneously: teachers might consider designing competence-supportive lessons with differentiated skill-progression tasks to address the facility deficit, while implementing cooperative peer structures, such as buddy systems and peer-mentoring programs, to cultivate the social connectedness that non-athletes lack outside school (Ntoumanis, 2001; Standage et al., 2005). Such approaches may help address the relational and environmental deficits identified as primary barriers more effectively than individual behavior change campaigns alone.

The finding that only 9.3% of non-athletes cited lack of time suggests that, in the urbanized context of Riyadh, a built-environment deficit, where safe, accessible sports facilities are insufficiently available outside school hours, may be a more significant constraint than time availability alone. It should be noted, however, that the ATLS questionnaire did not include a dedicated item for academic workload, and ‘lack of time’ cannot be interpreted as a direct proxy for academic pressure. Time constraints may reflect a range of competing factors, including commuting distance to sports facilities, family obligations, or other priorities. Indeed, it is plausible that some non-athletes possess sufficient time for both academic work and PA but are deterred by the distance or inaccessibility of suitable facilities, suggesting that proximity and accessibility may interact with perceived time availability in ways the current instrument was not designed to disentangle. The school PE setting may therefore assume strategic importance as a guaranteed, equitable PA opportunity that transcends the socioeconomic inequalities restricting access to community sports infrastructure (Sallis et al., 2012). Policy responses might consider prioritizing school sports facility development, expanding PE curriculum time, and establishing structured peer-based PA programs as approaches likely to be more effective than those focused solely on individual behavior change.

These findings warrant broader contextualization within Saudi Arabia’s rapidly evolving social and environmental landscape. Riyadh’s accelerated urbanization has produced a built environment in which residential areas are predominantly car-dependent, public open spaces are limited, and purpose-built sports facilities are unevenly distributed across socioeconomic zones (Al-Hazzaa, 2018; Khalaf et al., 2013). In this context, the dominance of structural and social barriers, rather than motivational or academic ones, may reflect a generation of adolescents who are willing to be active but lack the environmental and social infrastructure to do so. This pattern contrasts markedly with findings from Western contexts, where time constraints and academic pressure dominate (Allison et al., 1999; Pandolfo et al., 2016), and aligns more closely with findings from other rapidly urbanizing middle-income settings where built-environment deficits similarly constrain adolescent PA (Muhyi et al., 2024). Culturally, the finding that social barriers, specifically the absence of an exercise partner, ranked second among non-athletes may reflect the collectivist social norms prevalent in Saudi society, where physical activity is more naturally pursued within peer or family group contexts rather than individually. From a policy perspective, these findings suggest that effective interventions must operate simultaneously at multiple levels: investing in accessible community sports infrastructure, embedding PA opportunities within the compulsory school day, and fostering peer-based social structures that align with the social norms governing adolescent life in Saudi Arabia.

Several limitations of this study should be acknowledged. First, the cross-sectional design precludes causal inference regarding the relationship between PA participation and the observed health outcomes. Second, the sample included only male adolescents from a single Saudi Arabian city, thus limiting generalizability to female adolescents, younger age groups, and other regions of Saudi Arabia. Third, the classification of participants into the athlete and non-athlete groups was based on self-report data, thus introducing potential social desirability bias and inaccurate reporting due to recall bias (Klesges et al., 1990). Fourth, as lifestyle behavior data (screen time, sleep, diet) were self-reported, they may also have been subject to recall and social desirability biases (Coughlin, 1990). Fifth, neglecting to collect socioeconomic data on the participants may have skewed the results, as access to sports facilities and dietary choices are likely influenced by household income. To overcome these limitations, future studies should employ objective PA measurement (e.g., accelerometry), include female participants, and incorporate socioeconomic indicators, and extend the school-based focus by examining how specific PE teaching practices and school sport policies moderate the relationship between perceived barriers and PA engagement among Saudi adolescents (Alaqil et al., 2024; Evenson et al., 2023). Furthermore, the lifestyle variables assessed in this study were measured at a general level. Screen time was captured as total hours without distinguishing how that time was used, for example, educational, passive, or social media consumption, and dietary habits were assessed by consumption frequency rather than nutritional composition or overall dietary quality. Similarly, sleep was measured by duration alone, without capturing quality or consistency. Future studies should employ more granular measures of these variables, such as screen use diaries, validated dietary assessment tools, and sleep quality indices, to provide deeper insight into the lifestyle patterns of Saudi adolescents and their relationship with PA engagement.

## 5. Conclusions

The current study indicates a significant health disparity between athletic and non-athletic male adolescents in Riyadh, with non-athletes exhibiting substantially higher rates of overweight and central obesity. Crucially, the principal barriers to PA participation among non-athletes were structural and social, specifically, the absence of suitable exercise facilities and exercise partners, rather than academic time pressure, a finding that diverges markedly from the global literature. Interpreted through SDT, these barriers reflect unmet needs for competence and relatedness, both of which the school PE environment may be well positioned to address.

These findings have possible implications for PE teachers. Rather than focusing on infrastructure investment, PE educators might consider cultivating the intrinsic motivational profile, grounded in health and recreation, observed among the athletic subgroup, and intentionally extend it to non-athletes through need-supportive teaching practices. Concretely, this means designing competence-building lessons with differentiated skill-progression tasks and embedding cooperative peer structures such as buddy systems and group activities that foster social connectedness within the PE class. Such approaches may help address the relational and environmental deficits identified as the primary barriers in this population and may be more likely to promote long-term PA adherence than individual behavior change campaigns alone. Achieving this goal is both clinically urgent and aligned with the health and quality-of-life objectives of Saudi Vision 2030.

## Figures and Tables

**Table 1:** Anthropometric characteristics of participants.

Variable	Non-Athletes (*n* = 54)	Athletes (*n* = 70)	t-Value	*p*-Value
Age (years)	16.81 ± 0.73	16.77 ± 0.60	0.32	0.753
Height (m)	1.72 ± 0.07	1.76 ± 0.06	−3.12	0.002
Weight (kg)	87.51 ± 21.31	68.73 ± 15.10	5.13	<0.001
BMI (kg/m^2^)	29.40 ± 6.77	22.19 ± 4.44	6.51	<0.001
Waist circumference (cm)	98.65 ± 21.63	78.84 ± 9.51	5.88	<0.001

Value means ± standard deviations. BMI = body mass index. *p*-values derived from independent sample *t*-tests.

**Table 2:** Comparison of lifestyle behaviors.

Variable	Non-Athletes (*n* = 54)	Athletes (*n* = 70)	t-Value	*p*-Value
Screen Time Weekday (h)	2.41 ± 1.45	2.15 ± 1.39	1.00	0.316
Screen Time Weekend (h)	3.11 ± 1.54	2.97 ± 1.58	0.49	0.628
Sleep Weekday (h)	6.10 ± 1.71	6.30 ± 1.66	−0.66	0.511
Sleep Weekend (h)	7.45 ± 1.43	7.52 ± 1.50	−0.26	0.798
Breakfast Frequency (days/wk)	4.61 ± 2.96	5.19 ± 3.03	−1.08	0.280

Value means ± standard deviations. *p*-values derived from independent sample *t*-tests.

**Table 3:** Primary motivations for physical activity among athletes (*n* = 70).

Motivation	*n*	%	Motivation	*n*
Health	19	27.1%	Health	19
Recreation	18	25.7%	Recreation	18
Competition	14	20.0%	Competition	14
Weight Loss	9	12.9%	Weight Loss	9
Socializing	6	8.6%	Socializing	6

Frequency represents the number of participants selecting each response. Percentages may not sum to 100% due to rounding.

**Table 4:** Perceived barriers to physical activity among non-athletes (*n* = 54).

Barrier	*n*	%	Barrier	*n*
No suitable place available	14	25.9%	No suitable place available	14
No partner to exercise with	12	22.2%	No partner to exercise with	12
Health conditions	10	18.5%	Health conditions	10
Other reasons	9	16.7%	Other reasons	9
Lack of time	5	9.3%	Lack of time	5

Value means ± standard deviations. *p*-values derived from independent sample *t*-tests.

## Data Availability

The data are available from the corresponding author upon reasonable request.
